# The Clinical Significance of the Manchester Colour Wheel in a Sample of People Treated for Insured Injuries

**DOI:** 10.3390/jcm15010075

**Published:** 2025-12-22

**Authors:** John Edward McMahon, Ashley Craig, Ian Douglas Cameron

**Affiliations:** John Walsh Centre for Rehabilitation Research, Kolling Institute for Medical Research, School of Health Sciences, Faculty of Medicine and Health, The University of Sydney, St. Leonards, NSW 2065, Australia; a.craig@sydney.edu.au (A.C.); ian.cameron@sydney.edu.au (I.D.C.)

**Keywords:** Manchester Colour Wheel, machine learning, compensable injury, Whiplash Associated Disorder, back injury, shoulder injury

## Abstract

**Background/Objectives**: The Manchester Colour Wheel (MCW) was developed as an alternative way of assessing health status, mood and treatment outcomes. There has been a dearth of research on this alternative assessment approach. The present study examines the sensitivity of the MCW to pain, psychological factors and recovery status in 1098 people with insured injuries treated in an interdisciplinary clinic. **Methods**: A deidentified data set of clients treated in a multidisciplinary clinic was conveyed to the researchers, containing results of MCW and injury-specific psychometric tests at intake, as well as recovery status at discharge. Systematic machine modelling was applied. **Results**: There were no significant differences between the four injury types studied: motor crash-related Whiplash Associated Disorder (WAD) and workplace-related Shoulder Injury (SI), Back Injury (BI) and Neck Injury (NI) on the MCW. Augmenting the MCW with Machine Learning (ML) models showed overall classification rates for Classification and Regression Tree (CRT) of 75.6% for Anxiety, 70.3% classified for Depression and 68.5% for Stress, and Quick Unbiased Efficient Statistical Trees could identify 68.5% of Pain Catastrophisation and 62.7% of Kinesiophobia. Combining MCW with psychometric measurements markedly increased the predictive power, with a CRT model predicting WAD recovery status with 80.7% accuracy, SI recovery status 81.7% accuracy and BI recovery status with 78% accuracy. A Naïve Bayes Classifier predicted recovery status in NI with 96.4% accuracy. However, this likely represents overfitting. **Conclusions**: Overall, MCW augmented with ML offers a promising alternative to questionnaires, and the MCW appears to measure some unique psychological features that contribute to recovery from injury.

## 1. Introduction

Injury rehabilitation questionnaires are used to inform about the needs and likelihood of recovery and to personalise treatment. Cattel [1] makes the point that, although an ancient approach, asking questions is not necessarily a reliable method of assessment and criticises psychology because other, more novel methods may have greater utility. The Manchester Colour Wheel (MCW) was developed in 2010 as an alternative way of assessing health status, mood and treatment outcomes compared against standard psychometric tests [2]. It has been applied along with questionnaires to predict the recovery of people with irritable bowel syndrome [3], but there have been few other clinical applications of this measure. It has been validated with adults and adolescents [4]. This research shows that depressed adults and adolescents prefer dark shades, so-called “negative colours”, with black being the most favoured colour. The studies on the MCW showed that this mood colour preference was subject to change in the short term after interventions. In a multilingual society such as Australia, a valid and reliable measure that is minimally dependent on English-language comprehension, has simple instructions, is easily translatable and has less time burden than questionnaires would improve clinical practice.

Variations in the wording of the MCW were noted to improve its sensitivity to mood disorders [5]. Mood disorders have been associated with changes in the subjective report of colours with a decrease in colour sensitivity [6]. People associate different colours with different attitudes and emotions, and they may also have cultural connotations and associations [7]. Imagery and colour are important factors in approaches to treating psychosomatic conditions where there is a psychological overlay contributing to the experience of the physical condition, or complicating recovery from the condition. Recent functional Magnetic Resonance Imaging (fMRI) research has demonstrated that there are uniform patterns of neural activation in response to the same colour presented to a different brain, suggesting a uniform perceptual process resulting from common functional or evolutionary pressures [8]. Written descriptions of colour, such as the word “red” or “blue”, can have different emotional responses to visual presentations of colour patches in a cohort of Chinese people, indicating different associative pathways for written and visual presentation [9]. This finding has been replicated with research on the colours from the Geneva Colour Wheel, which demonstrated similar emotional associations between visual presentation of colour and colour words [10]. A systematic review of 132 papers, encompassing 42, 266 people spanning 64 countries, showed basic colour categories associated with specific emotions, showed colour saturation associated with negativity and intensity of emotion and showed colours are similarly associated with colours across cultures [11]. Pain is a ubiquitous human experience; however, a broad narrative review of pain expression, communication and pain coping across ethnic and cultural backgrounds shows marked differences [12]. This indicates a need to have a nuanced approach to assessing the experience of pain. The use of the MCW to communicate pain and the emotional responses associated with injury may enhance its treatment.

Both hypnosis [13,14] and eye movement desensitisation and reprocessing therapy (EMDR) [15] use the mental features of colour and image as targets for change to transform the injured person’s experience of their condition. This imagery has been shown to change with treatment of conditions, including irritable bowel syndrome [16] and chronic pain [17]. The verbal or written suggestions made about colour selection in the MCW are important to how colour and imagery are related to by the individual, and these can be altered by therapeutic interventions.

The relationship between the left and right hemispheres of the brain and their roles in emotion and trauma is a nuanced topic that has been extensively studied [18]. The lateralisation of brain functions indicates that each hemisphere contributes differently to these processes [19]. Historically, the right hemisphere is predominantly associated with the processing of emotions, particularly negative emotions such as fear and sadness. This hemisphere is also linked to the vividness and intensity of traumatic memories, mediates the retrieval of autobiographical memories and regulates body states, making it particularly relevant in trauma-related disorders [20,21]. The left hemisphere is more involved in language and analytical processing, playing a significant role in the regulation and expression of positive emotions and the construction of the narrative of a person’s life [22]. It contributes to the cognitive appraisal and verbalisation of emotional experiences, which is particularly important in therapeutic contexts where verbal communication is essential, and also the interpretation and self-appraisal required in the answering of questionnaires. The interaction between the hemispheres is critical for balanced emotional processing. Recent research suggests that emotional processing involves multiple interrelated networks, each associated with different components of emotion generation, perception and regulation [23].

Machine Learning (ML) and Artificial Intelligence have been applied to a multitude of medical problems, including imaging and diagnosis [24,25], triage and recovery prediction [26,27,28,29,30,31,32] and link diverse data, such as newer research on Posttraumatic Stress Disorder (PTSD) [33,34,35] and on chronic pain [36]. Gozzi et al. [37] used Machine Learning models to show that patients with high subjective reported pain relative to physiological variables were characterised by long-term disability, poor recovery and distress expressed as Anxiety and Depression.

According to Safework Australia, the most common work-related injuries are the upper limbs, trunk, lower limbs, non-physical injuries and head and neck injuries (NIs) [38]. According to the New South Wales (NSW) State Insurance Regulatory Authority, the most common motor vehicle-related injury is Whiplash Associated Disorder (WAD) [39]. Digital Intervention (DI) has been demonstrated to be as effective as treatment as usual for chronic back pain [40]. The Active Recovery Clinics (ARC) were developed to treat high-frequency physical injuries that respond to active treatment by augmenting an interdisciplinary clinic with DI to ensure exercise adherence. SWORD Phoenix is a DI system that uses a tablet to demonstrate exercises to clients and sensors to monitor motion and give immediate feedback on exercise performance and compliance [41]. This DI has been used in the successful treatment of chronic shoulder pain [42], chronic lower back pain [43] and hip pain [44] and in telerehabilitation of acute musculoskeletal conditions [45]. The ARC combines this DI system with assessment and interdisciplinary oversight by a medical specialist, psychologist and physiotherapist. Anxiety and Depression are associated with poor active program adherence [46] and generally with poor outcomes in rehabilitation of injury. In ARC, the psychosocial barriers to recovery, including Anxiety, Depression and accident- and injury-related trauma symptoms, are addressed with the psychological intervention of EMDR.

Through retrospective data mining use of ML to derive insights, the first aim of this study was to evaluate and compare the recovery dynamics in the interdisciplinary treatment of WAD, Shoulder Injury (SI), Back Injury (BI) and NI. The second aim was to see if the MCW has sensitivity to factors important to adjustment to injury, including pain, Depression, Anxiety, Stress, Kinesophobia and Pain Catastrophising relative to an established question protocol [46]. A further aim of this study is to identify the questions that lead to clinically meaningful responses on the MCW for people with musculoskeletal injuries presenting for treatment and developing ML systems for interpretation of the MCW. Finally, the identification of prognostic indicators will be used to derive insights into the dynamics that underpin these injuries, potentially integrating the left-hemisphere verbally mediated sense of injury and the right-hemisphere visuospatial-mediated sense of injury.

## 2. Materials and Methods

### 2.1. Participants

Participants were 1098 personal injury claimants who had suffered injury in the course of their work (*n* = 827) or in motor vehicle crashes (*n* = 271) who opted into treatment with the Active Recovery Clinic (ARC) when it was offered by the insurer or scheme agent. The research period was from 1 January 2022 to 8 February 2024. The mean age was 42.4 years (SD = 13.3, range = 18–74), 51.9% were female, 2.7% were unemployed or retired, 0.6% were students, 10.1% were in professions, 11.4% were frontline workers in health or law enforcement and the remaining 75.27% were in general employment. Participants were reviewed at initial onboarding at clinic locations across Australia. The participants suffered Back injury (BI, *n* = 359, 32.7%), Shoulder Injury (SI, *n* = 391, 35.6%), Neck Injury (NI, *n* = 59, 5.4%) and Whiplash Associated Disorder (WAD, *n* = 287, 26.1%). Two clients were excluded for non-specific injuries (*n* = 2, 0.02%). All clients spoke English sufficiently to complete the questionnaires. Clients were deemed “Unsuitable for Clinic (USC)” if they had complicating injuries or medical conditions, which warranted more intensive intervention or prevented engagement in active rehabilitation. Those who required surgery were coded as “Surgical Withdrawals (SW)”. Those who attended assessment but opted out of the program during the onboarding process were coded as “did not commence (DNC)”. Those for whom the insurer declined a claim after referral were coded as “Administrative Withdrawals (AW)”. Clients who commenced the program and did not complete were coded as “did not complete (DNCP). Clients were deemed “fully recovered (FR)” if there were no clinically significant symptoms and they were certified fit to return to pre-injury duties, “partially recovered (PR)” if there was improvement in symptoms and return to restricted duties and “no recovery (NR)” if there was no clinically significant improvement in their condition and no return to duties.

### 2.2. Procedure

The ARC programs are technology-augmented interdisciplinary clinics of up to 12 weeks in duration. All note-keeping and data were held in an instance of Salesforce Health Cloud, which was warehoused locally. The programs consisted of reviews by an interdisciplinary panel consisting of an orthopaedic surgeon or sports medicine physician, a psychologist and a physiotherapist, at onboarding and weeks 4, 8 and 12. Prior to each clinic attendance, clients completed a brief psychometric screening consisting of Vaegter’s screening questions [46], the Visual Analogue Pain Scale (VAS) [47], and the MCW [2] administered by secure portal interface via the client’s smart device. Client’s optimism about recovery was assessed by asking if they thought they were going to get better with responses “yes”, “unsure” and “no”. Each professional spent 30 min with each participant at each review. The medical examination involved a general history-taking of the injury and medical background; medical assessment of the injury, including range of motion and symptoms; and an orientation to the interdisciplinary program. The psychological assessment involved a semi-structured clinical interview inquiring about history of mental disorder, previous trauma exposure and assessment of the presenting issue. Clients with significant psychological trauma or symptoms were provided with psychological therapy by way of EMDR by “Weekend 2” certified practitioners [48]. The physiotherapy review included an assessment of physical capacity and orientation to and configuration of the SWORD Phoenix exercise system with a program of exercises from a pre-programmed catalogue customised to the client’s injury and goals. The exercises for BI and SI were prescribed from the SWORD Phoenix library [40,41,42,43,44,45] of exercises. For BI, this was in combination with lower-limb strengthening exercises, if required, for fear of reinjury and return-to-work hardening [49]. The catalogue of exercises for WAD and NI was taken from the State Insurance Regulatory Authority Guidelines [39] for the management of Acute Whiplash Associated Disorders, which were generated and ported into the SWORD Phoenix Platform. A de-identified database was created and supplied to the researchers by the Head of Data and Analytics of Navigator Group.

### 2.3. Measures

#### 2.3.1. Pain

The VAS [47] was administered to assess pain and has been validated for use on digital platforms [50]. There have been attempts to develop cutoffs for “mild”, “moderate” and “severe pain” with systems by Jensen et al. [51] and Boonstra et al. [52]. Three different sets of cutoffs were applied to compare the utility and predictive validity of the cut-points. Clinically significant differences on the VAS were considered 12 mm [42] or greater than 33% [44].

#### 2.3.2. Psychological Distress

Vaegter et al. [46] developed a 6-item screening questionnaire based on the closed-ended items rated from 0 to 10, with classifying cut-points on single items for clinically significant symptoms of Anxiety (SE = 80.8%, SP = 72.2%), Stress (SE = 71.2%, SP = 70.6%), Pain Catastrophisation (SE = 90.7%, SP = 60.9%), or with Kinesiophobia (SE 75.7%, SP = 74.4%); and with two items to classify significant Depression (SE 77.2%, SP = 73.1%). The items were derived from the Generalized Anxiety Disorder Scale [53], the Perceived Stress Scale [54], the Pain Catastrophisation Scale [55], the Tampa Scale for Kinesiophobia [56], and the Patient Health Questionnaire-9 [49], respectively. These were the minimum number of items to separate between people classified by each of these scales as being significantly elevated on the test construct or not. This measure was used to classify the presence of these psychological features in presenting clients.

#### 2.3.3. Optimism About Recovery

The question “Do you think you are going to get better?” was rated by participants as “yes”, “unsure” and “no” to assess optimism about recovery, with optimism being considered “yes”.

#### 2.3.4. Manchester Colour Wheel

The MCW requires the examinee to rate the test construct on the basis of 38 colours that vary in hue, tint, shade and tonality. It consists of six hues: red, orange, yellow, green, blue and purple, with 4 gradations of shade, and 6 tints, from black through greys to white. Research showed test–retest reliability for “drawn to”, “favourite” and “mood” colour over 2 weeks [2]. In early research, the colours of the MCW were clustered into eleven “Positive”, eighteen “Neutral”, and nine “Negative” colours [3]; hereafter, these are referred to as “Permutation 0”. Later research [2] developed a further 8 colour permutations of positive, neutral and negative colours with varying sensitivity to mood and interest, with increasing restriction on the positive and negative ends of the spectrum. Permutations 5 and 6, and Permutations 7 and 8 were collapsed together due to significant redundancy [2]. The individual colours and permutation categories were used to interpret the colour choice. Participants were asked to respond on the MCW to the following questions:

1. Which colour do you feel most drawn to?
2. What colour is your favourite colour?
3. What colour represents your day-to-day mood?
4. What colour represents your day-to-day pain?

### 2.4. Software and Data Analysis

IBM SPSS 28 was used to calculate all statistics. Chi-Square Tests were used to compare groups on categorical variables such as optimism about recovery, the MCW colours and permutations of colours. Cramer’s V was used to calculate effect size. Given the uneven samples sizes and non-normality of variance, Kruskal–Wallis H-tests were used to compare continuous and ordinal variables between groups with 2-sided asymptotic significance, and Bonferroni-adjusted significance for multiple Pairwise Comparisons was used for significance testing. While means and standard deviations are reported, given the small size of the AW group (*n* = 5), these were removed from the latter analyses, and the SW group was collapsed into the USC group for analysis to increase power for recovery results. ANOVA was used to compare pre- and post-testing within groups that participated in treatment (FR, PR, NR). Given the presence of “0” endorsement on cells in the MCW selections for the different injuries, Monte Carlo Simulations were employed alongside the Chi-Square Test to generate approximate *p*-values where indicated [57]. Given the size of the matrices for calculation (4 × 38, 2 × 38), it was computationally infeasible to use a Fischer’s Exact calculation. Machine Learning models of Chi-square Automatic Interaction Detector (CHAID [58]), Exhaustive CHAID (Ex-CHAID), Classification and Regression Tree (CRT [59]) analysis, and Quick Unbiased Efficient Statistical Tree (QUEST [60]) algorithms and Naïve Bayesian Classifier (NBC [61]) models were generated to explore relationships between variables and to operationalise the interpretation of the MCW on 80% training data and 20% testing data. Macro Area Under the Curve (Macro AUC), an average of the area under the classification curves, as well as Specificity (Sp) and Sensitivity (Sn), were calculated for the best predictive models. Julius.AI was used to generate the figures displayed below.

## 3. Results

### 3.1. Clinical Findings

#### 3.1.1. Onboarding Statistics

Examining the onboarding scores of outcome groups showed multiple significant differences in personal and psychological characteristics. Table 1 contains the clinical results from those initially referred at the time of onboarding. The Kruskal–Wallis H-Test showed there was a significant difference in age across the outcome groups, χ^2^ (df = 7, *N* = 1015) = 39.13, *p* < 0.001. Pairwise comparison, with adjusted Bonferroni correction for multiple tests, showed that the Surgical Withdrawal group was older than the DNCP (ρ = 0.031), DNC (ρ = 0.14) and FR (ρ = 0.08) groups. The Kruskal–Wallis H-Test showed a significant difference in Injury to Onboard Interval, χ^2^ (df = 7, *N* = 1096) = 17.91, ρ = 0.012. Pairwise comparison showed that PR had longer injury to onboard intervals than FR, but this only approached significance on post hoc testing (ρ = 0.061). The Kruskal–Wallis H-Test showed significant differences in VAS scores between outcome groups, χ^2^ (df = 7, *N* = 1096) = 153.24, ρ < 0.001. Pairwise comparison showed that USC (ρ = 0.003), PR (ρ < 0.001), DNC (ρ < 0.001), NR (ρ = 0.019) and DNCP (ρ < 0.001) were significantly higher than FR, and using a 12 mm reliable change indicator [42], those with FR had clinically significantly less pain. The Kruskal–Wallis H-Test showed significant differences in Stress scores between outcome groups, χ^2^ (df = 7, *N* = 1096) = 76.62, ρ < 0.001. Pairwise comparison showed that USC (ρ = 0.004), PR (ρ < 0.001), DNC (ρ < 0.001), NR (ρ = 0.019) and DNCP (ρ < 0.001) were significantly more stressed than FR. The Kruskal–Wallis H-Test showed significant differences in Anxiety scores between outcome groups, χ^2^ (df = 7, *N* = 1096) = 53.52, ρ < 0.001. Pairwise comparison showed that PR (ρ < 0.002), DNC (ρ < 0.001), NR (ρ = 0.019) and DNCP (ρ < 0.001) were significantly more anxious than FR. The Kruskal–Wallis H-Test showed significant differences in Hopelessness scores between outcome groups, χ^2^ (df = 7, *N* = 1096) = 76.67, ρ < 0.001. Pairwise comparison showed that PR (ρ < 0.001), DNC (ρ < 0.001), NR (ρ = 0.001) and DNCP (ρ < 0.001) were significantly more hopeless than FR. The Kruskal–Wallis H-Test showed significant differences in Low Interest scores between outcome groups, χ^2^ (df = 7, *N* = 1096) = 69.53, ρ < 0.001. Pairwise comparison showed that PR (ρ = 0.001), DNC (ρ < 0.001), NR (ρ = 0.001) and DNCP (ρ < 0.001) were bothered by Low Interest compared to FR. The Kruskal–Wallis H-Test showed significant differences in Pain Catastrophising scores between outcome groups, χ^2^ (df = 7, *N* = 1096) = 102.25, ρ < 0.001. Pairwise comparison showed that PR (ρ < 0.001), DNC (ρ < 0.001), NR (ρ = 0.010) and DNCP (ρ < 0.001) had significantly more Pain Catastrophising than FR. The Kruskal–Wallis H-Test showed significant differences in Kinesiophobia scores between outcome groups, χ^2^ (df = 7, *N* = 1096) = 91.36, ρ < 0.001. Pairwise comparison showed that PR (ρ < 0.001), DNC (ρ < 0.001), SW (ρ < 0.001), PR (ρ < 0.001), DNCP (ρ < 0.001) and NR (ρ < 0.001) had significantly more Kinesiophobia than FR, and that DNC (ρ < 0.001) had more Kinesiophobia than PR. The Chi-Square Test (χ^2^ = 116.388, ρ < 0.001) revealed significant differences between groups on outcomes for optimism about recovery, with those in FR more optimistic about recovery than other groups, with a medium effect size (Cramer’s V = 0.23, ρ < 0.001). ANOVA revealed no significant differences between pre- and post-intervention testing on psychometric measures for NR, PR and FR.

#### 3.1.2. Onboarding Statistics by Injury Types

Table 2 contains the means and standard deviations at onboarding of the four program groups. On comparing the clinical groups, the Kruskal–Wallis H-Test showed there was a significant difference in Age between program types, χ^2^ (df = 3, *N* = 1015) = 26.75, ρ < 0.001. Pairwise comparison showed that WAD (ρ < 0.001) and BI (ρ = 0.001) were younger than SI. The Kruskal–Wallis H-Test showed there was a significant difference in Injury to Onboard Interval between program types, χ^2^ (df = 3, *N* = 1096) = 87.69, ρ < 0.001. Pairwise comparison showed that WAD Injury to Onboard Interval was shorter than BI (ρ < 0.001), NI (ρ = 0.001) and SI (ρ = 0.001). The Kruskal–Wallis H-Test showed there was a significant difference between program types on the VAS, χ^2^ (df = 3, *N* = 1096) = 8.42, ρ = 0.038. Pairwise Comparison showed the WAD rating to be higher than SI (ρ = 0.031) in the pain rating. The Kruskal–Wallis H-Test showed there was a significant difference between program types for Stress, χ^2^ (df = 3, *N* = 1096) = 27.38, ρ < 0.001. Pairwise comparison showed BI (ρ = 0.009), NI (ρ = 0.004) and WAD (ρ < 0.001) were more stressed than SI. The Kruskal–Wallis H-Test showed there was a significant difference between program types on Anxiety, χ^2^ (df = 3, *N* = 1096) = 24.426, ρ < 0.001. Pairwise comparison showed WAD (ρ < 0.001) were more anxious than SI. The Kruskal–Wallis H-Test showed there was a significant difference between program types on Hopelessness, χ^2^ (df = 3, *N* = 1096) = 23.607, ρ < 0.001. Pairwise comparison showed that WAD (ρ < 0.001), NI (ρ < 0.035) and BI (ρ = 0.005) were more hopeless than SI. The Kruskal–Wallis H-Test showed there was a significant difference between program types on Pain Catastrophising, χ^2^ (df = 3, *N* = 1096) = 9.000, ρ = 0.29. Pairwise comparison showed NI was more Pain Catastrophising than SI. The Chi-Square Test (χ^2^ = 6.259, ρ = 0.395) revealed no difference between programs on optimism about recovery.

#### 3.1.3. Program Outcomes by Injury Type

Table 3 contains the outcomes by injury type. The Chi-Square Test (χ^2^ = 51.729, *N* = 1091, df = 15) ρ < 0.001) showed significant differences between groups on outcomes on recovery status, with a small effect size (Cramer’s V = 0.115, ρ < 0.001). NI and SI had more participants found not suitable for the clinic compared to WAD and BI. Inspection showed more SW in SI than in the other groups. NI had fewer DNC than other groups but had more DNCP. SI had more FR compared to other groups and a lower percentage of PR.

### 3.2. Construct Validity of the Manchester Colour Wheel

#### 3.2.1. Sensitivity to Injury Type

The Chi-Square Test enhanced with Monte Carlo Simulations showed no significant differences in colour selection between injuries in response to Colour Drawn To Colour (χ^2^ = 129.299, *N* = 1091, df = 111, ρ = 0.113), Favourite Colour (χ^2^ = 130.347, *N* = 1091, df = 111, ρ = 0.101), Day-to-Day Mood (χ^2^ = 103.319, *N* = 1091, df = 111, ρ = 0.686) or Day-to-Day Pain. However, Day-to-Day Pain approached significance (χ^2^ = 132.650, *N* = 1091, df = 111, ρ = 0.079) with a small effect size (Cramer’s V = 0.078, CI = 99%: Lower Bound 0.071, Upper Bound = 0.85), perhaps reflecting a pain differential between SI and WAD.

#### 3.2.2. Sensitivity to Pain Severity Classification

**Figure 1:**
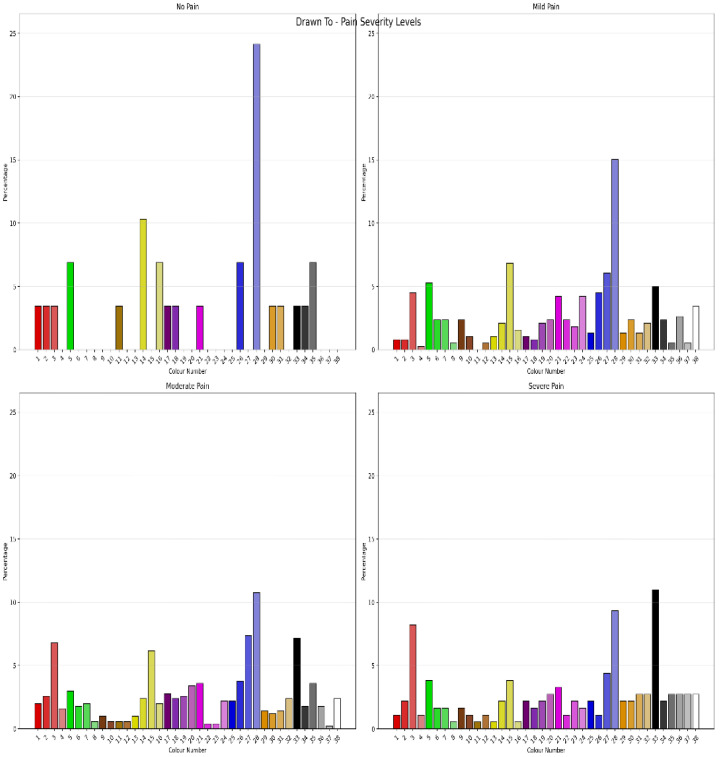
Percentage of “Drawn to” colour selections by pain severity classification.

**Figure 2:**
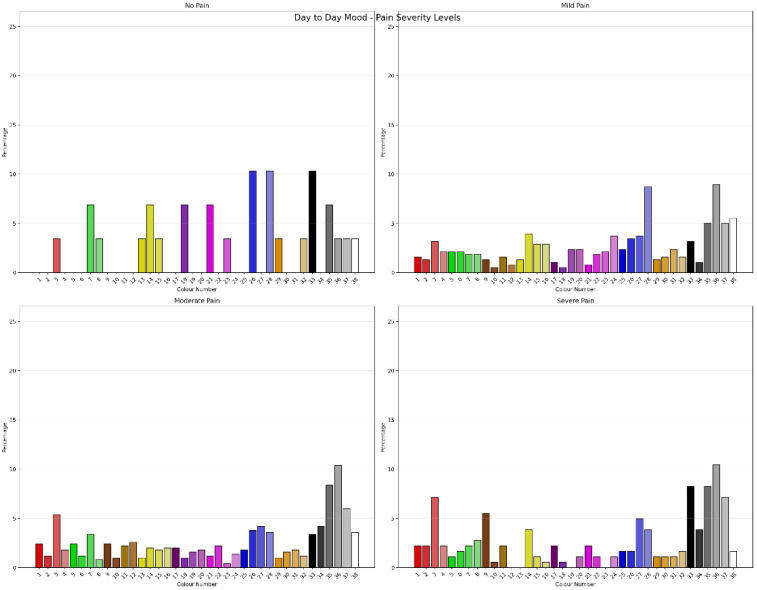
Percentage of “Day to Day Mood” colour selection by pain severity classification.

**Figure 3:**
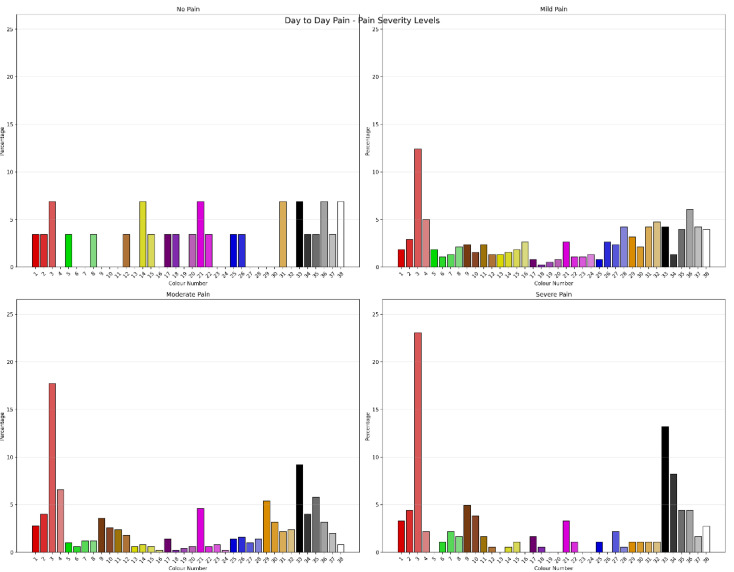
Percentage of “Day to Day Pain” colour selection by pain severity classification.

The Chi-Square Test showed that for clients with pain severity classified as “no pain”, “mild pain”, “moderate pain” and “severe pain”, using Jensen’s cut-scores showed significant differences in colour “Drawn To” (χ^2^ = 138.252, *N* = 1091, df = 111, *p* = 0.041), with a moderate degree of association (Cramer’s V = 0.041) between colour choice and pain severity. Figure 1 shows the percentage of “Drawn To” Colour choices by pain severity classification. This was equivalent to the Monte Carlo Simulation (CI = 99%: Lower Bound = 0.041, Upper Bound = 0.052). Pain severity classification also showed significant differences in “Day-to-Day Mood” colour selection (χ^2^ = 159.970, *N* = 1091, df = 111, *p* = 0.002) with a moderate degree of association (Cramer’s V = 0.221, ρ = 0.002). This was equivalent to the Monte Carlo Simulation (CI = 99%: Lower Bound = 0.002, Upper Bound = 0.005). Figure 2 shows the percentage of “Day to Day Mood” colour selection by pain severity classification. Pain severity classification also showed significant differences on colour selection for “Day-to-Day Pain” (χ^2^ = 202.753, *N* = 1091, df = 111, *p* < 0.001) with a moderate degree of association (Cramer’s V = 0.249, ρ < 0.001). This was equivalent to the Monte Carlo Simulation (CI = 99%: Lower Bound < 0.001, Upper Bound < 0.001). Figure 3 shows the percentage of “Day to Day Pain” colour selection by pain severity classification. Chi-Square analysis showed there were no differences between pain severity classification and “Favourite Colour”. Repeating the analysis with Boonstra et al.’s pain classification systems [52] did not improve or significantly change the results. Comparing the permutations of Positive, Negative and Neutral Colours as outlined by Carruthers et al. [2,3] for classifications of pain severity showed that the permutations had different sensitivities to pain severity. Favourite Colour showed no differences between pain severity; however, “Drawn to”, “Day-to-Day Mood” and “Day-to-day Pain” across permutations were different across pain severity classification levels.

Augmenting with ML models showed an NBC which classified Jensen Pain Severity Classification on the basis of Drawn To Colour with 57.8% accuracy on training. The best DTs on training were both CRT models, which used MCW ratings in order of contribution as follows: Day to Day Pain, Day to Day Mood, Favourite Colour and Drawn To Colour. On testing, an Ex-CHAID (5/2) model performed best and classified on the basis of Day to Day Pain only with “mild pain” rating colours greater than 35, and “moderate pain” rating pain as colours less than or equal to 11, and colours 31 and 35 with 49.5% accuracy, showing poor performance for discriminating pain intensity.

#### 3.2.3. Sensitivity to Anxious Classification

**Figure 4:**
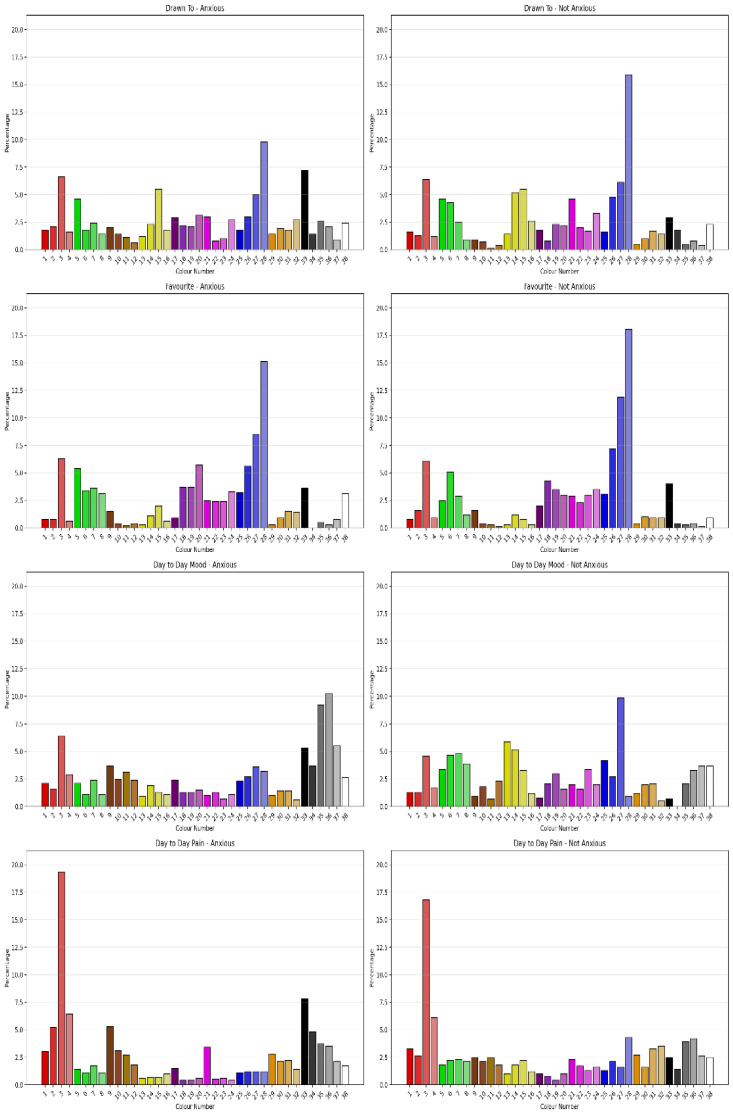
Percentages of colour selection for each question by anxious classification.

The Chi-Square Test showed that clients classified as Anxious using Vaegter’s [46] cut-score showed significant difference in Colour Drawn To Colour (χ^2^ = 73.202, *N* = 1091, df = 37, ρ < 0.001) with moderate degree of association (Cramer’s V = 0.259, ρ < 0.001, Eta = 0.249), Favourite Colour (χ^2^ = 54.379, *N* = 1091, df = 37, ρ = 0.033) with a moderate degree of association (Cramer’s V = 0.233, ρ = 0.033), Day to Day Mood (χ^2^ = 189.001, *N* = 1091, df = 37, ρ < 0.001) with strong association (Cramer’s V = 0.416, ρ < 0.001), and Day to Day Pain (χ^2^ = 102.643, *N* = 1091, df = 37, ρ < 0.001) with moderate association (Cramer’s V = 0.307, ρ < 0.001). Figure 4 shows the percentage of colour selection by Anxiety classification for the four questions. Comparing the permutations of Positive, Negative and Neutral Colours as outlined by Carruthers et al. [2,3] for sensitivity to Anxiety classification showed the permutations had different sensitivities to Anxiety. Favourite Colour Permutations 1 and 3 showed some sensitivity to Anxious classification; however, “Drawn to”, “Day-to-Day Mood” and “Day-to-day Pain” across permutations were sensitive to Anxious classification.

Augmenting with ML models showed the NBC model was the best, correctly classifying 78% of cases using Drawn To Colour in training. Of the DTs, a CRT (10/2) model classified 75.6% of cases on testing with 29 terminal nodes and a tree depth of 5 and used Favourite Colour; Day to Day Mood; Day to Day Pain; and Drawn To Colour. Table 4 contains the performance of the different models generated for Anxiety, Stress, Depression, Pain Catastrophisation and Kinesiophobia. Including the permutations made minimal improvement in classification. However, an Ex-CHAID model classified 77.6% of cases on testing with 11 terminal nodes and a tree depth of 3 and used Day to Day Mood Permutation 0; Drawn To Colour; Day to Day Pain Permutation 7 and 8; Day to Day Mood Permutation 1: Day to Day Mood; and Drawn To Permutation 2. Table 4 contains the performance of the different ML models generated, including permutations.

#### 3.2.4. Sensitivity to Stressed Classification

**Figure 5:**
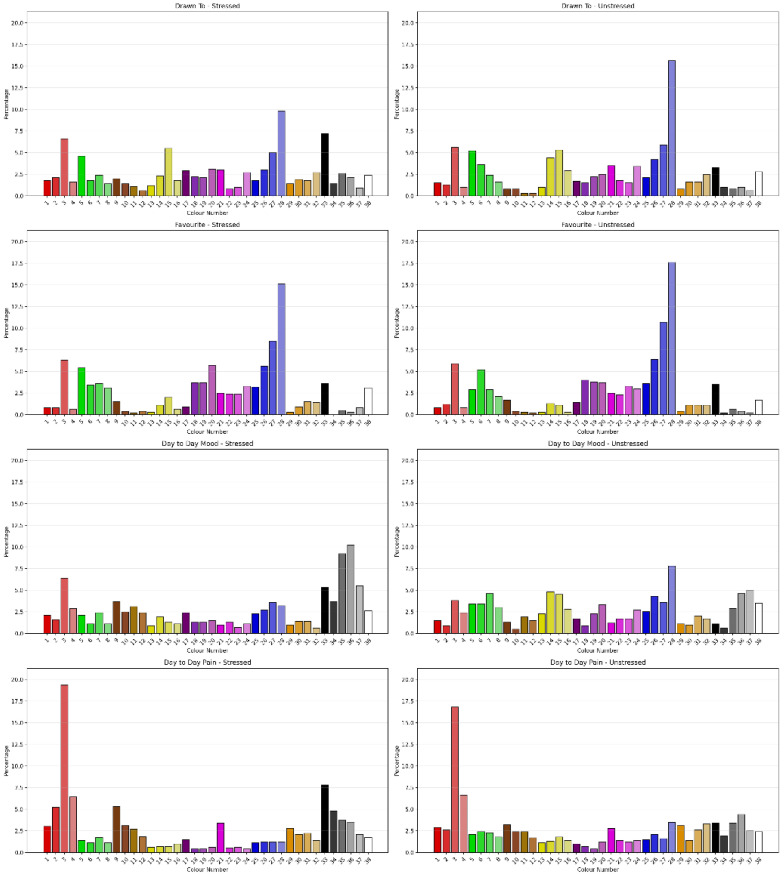
Percentages of colour selection for each question by Stressed classification.

The Chi-Square Test showed that clients classified as Stressed using Vaegter’s [46] cut-score showed significant difference in Drawn To Colour (χ^2^ = 86.038, *N* = 1091, df = 37, ρ < 0.001) with a moderate degree of association (Cramer’s V = 0.281, ρ < 0.001, Eta = 0.281), Favourite Colour (χ^2^ = 54.943, *N* = 1091, df = 37, ρ < 0.029) with a moderate degree of association (Cramer’s V = 0.224, ρ < 0.001, Eta = 0.224), Day to Day Mood (χ^2^ = 183.707, *N* = 1091, df = 37, ρ < 0.001) with a moderate-to-strong degree of association (Cramer’s V = 0.410, p < 0.001, Eta = 0.410) and Day to Day Pain (χ^2^ = 98.818, *N* = 1091, df = 37, ρ < 0.001) with a moderate degree of association (Cramer’s V = 0.301, ρ < 0.001, Eta = 0.301). Figure 5 shows the percentage of colour selection for each question by Stressed classification. Comparing the permutations of Positive, Negative and Neutral Colours as outlined by Carruthers et al. [2,3] for sensitivity to Stressed classification showed the permutations had different sensitivities to Anxiety severity. Favourite Colour Permutation 1 showed some sensitivity to Stressed classification; however, Drawn To, Day-to-Day Mood and Day-to-Day Pain across permutations were sensitive to Stressed classification.

Augmentation with ML models showed NBC was best on training, correctly classifying 74.1% of cases. The best DT model was a CRT (5/2) model, which classified 72.0% on training classifying with 20 terminal nodes and a tree depth of 5. However, on testing, an Ex-CHAID model identified 71.6% of cases with 16 terminal nodes and tree depth of 3 and used Day to Day Mood Permutation 1; Day to Day Mood Permutation 2; Day to Day Mood; Day to Day Pain Permutation 5 and 6, 7 and 8; Favourite Colour; and Day to Day Pain.

#### 3.2.5. Sensitivity to Depressed Classification

**Figure 6:**
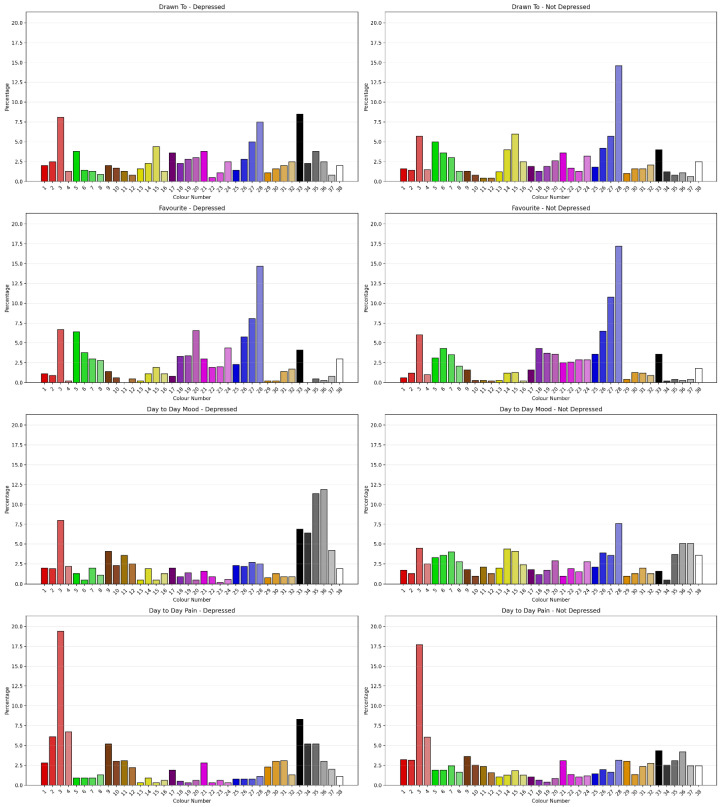
Percentage of colour selection for each question by Depressed classification.

The Chi-Square Test showed that clients classified as depressed using Vaegter’s [46] cut-score showed significant difference in Drawn To Colour (χ^2^ = 79.419, *N* = 1091, df = 37, ρ < 0.001) with a moderate degree of association (Cramer’s V = 0.270, ρ < 0.001, Eta = 0.270), Day to Day Mood (χ^2^ = 203.154, *N* = 1091, df = 37, ρ < 0.001) with a moderate-to-strong degree of association (Cramer’s V = 0.432, *p* < 0.001, Eta = 0.410) and Day to Day Pain (χ^2^ = 90.779, *N* = 1091, df = 37, ρ < 0.001) with a small degree of association (Cramer’s V = 0.288, ρ < 0.001, Eta = 0.288). Figure 6 shows the percentage of colour selection for each question by Depressed classification. There was no significant difference for Favourite Colour. Comparing the permutations of Positive, Negative and Neutral Colours as outlined by Carruthers et al. [2,3] for sensitivity to Depressed classification showed the permutations had different sensitivities to Depression classification. Favourite Colour Permutation 1 showed some sensitivity to Depressed classification. “Drawn to”, “Day-to-Day Mood” and “Day-to-day Pain” across permutations were sensitive to Depressed classification.

Augmentation with ML showed that a CRT (5/2) Model based on Permutations and Colours was best on training, correctly classifying 75.4% of cases with 27 terminal nodes and a tree depth of 5. On testing, a CRT (5/2) Model based on colours was best with 27 terminal nodes and a tree depth of 5 using Day to Day Mood; Day to Day Pain; Drawn To Colour; and Favourite Colour.

#### 3.2.6. Sensitivity to Pain Catastrophisation Classification

**Figure 7:**
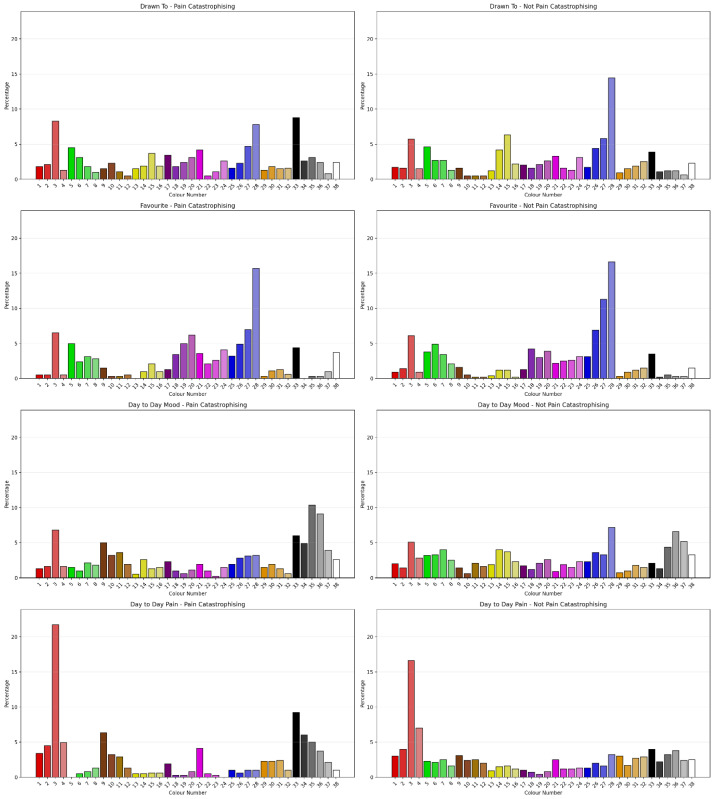
Percentage of colour selection for question by Pain Catastrophisation classification.

The Chi-Square Test showed that clients classified as Pain Catastrophising using Vaegter’s [46] cut-score showed a significant difference in Drawn To Colour (χ^2^ = 75.527, *N* = 1091, df = 37, ρ < 0.001) with a small to moderate degree of association (Cramer’s V = 0.256, ρ < 0.001, Eta = 0.256), Favourite Colour (χ^2^ = 53.278, *N* = 1091, df = 37, ρ < 0.001) with a small to moderate degree of association (Cramer’s V = 0.221, ρ = 0.041), Day to Day Mood (χ^2^ = 120.876, *N* = 1091, df = 37, ρ < 0.001) with a moderate degree of association (Cramer’s V = 0.333, ρ < 0.001, Eta = 0.333) and Day to Day Pain (χ^2^ = 104.204, *N* = 1091, df = 37, ρ < 0.001) with a moderate degree of association (Cramer’s V = 0.309, ρ < 0.001, Eta = 0.309). Figure 7 shows the percentage of colour selection for each question by Pain Catastrophising classification. Comparing the permutations of Positive, Negative and Neutral Colours as outlined by Carruthers et al. [2,3] for sensitivity to Pain Catastrophising classification showed the permutations had different sensitivities to Pain Catastrophising classification. Favourite Colour Permutation 1 showed some sensitivity to Pain Catastrophising classification. “Drawn to”, “Day-to-Day Mood” and “Day-to-day Pain” across permutations were sensitive to Pain Catastrophisation classification.

Augmenting with ML showed that in training, an NBC model correctly classified 74.6% of cases on the basis of Day to Day Mood. A QUEST (10/2) model was best on testing and correctly classified 69.4% of cases with 10 terminal nodes and a tree depth of 5 using, in order, Day to Day Pain, Day to Day Mood, Drawn To Colour and Favourite Colour.

#### 3.2.7. Sensitivity to Kinesiophobia Classification

**Figure 8:**
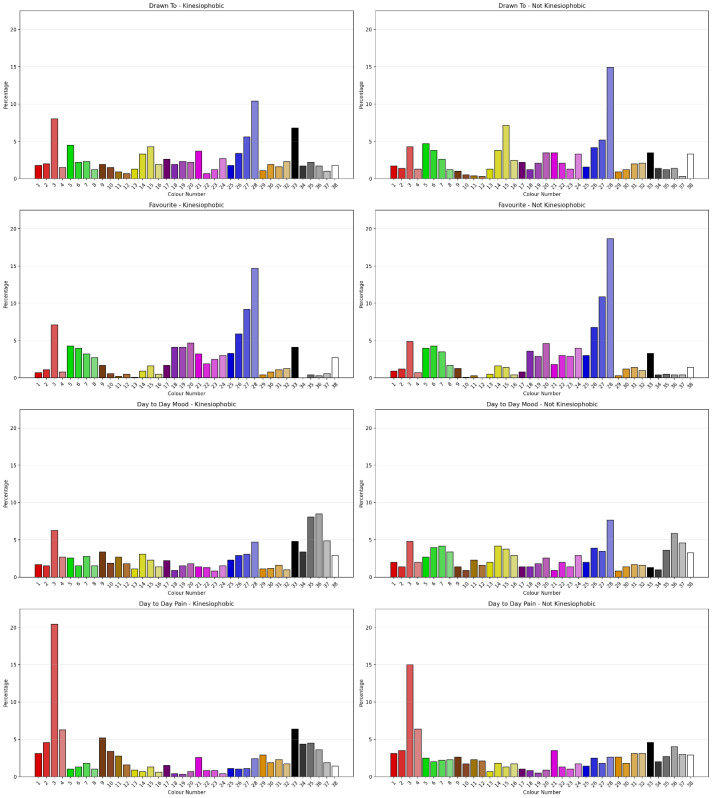
Percentages of colour selection for each question by Kinesiophobia classification.

The Chi-Square Test showed that clients classified as Kinesiophobic using Vaegater et al.’s [46] cut-score showed a significant difference in Drawn To Colour (χ^2^ = 57.807, *N* = 1091, df = 37, ρ = 0.016) with a small to moderate degree of association (Cramer’s V = 0.230, ρ = 0.016, Eta = 0.230), Day to Day Mood (χ^2^ = 79.066, *N* = 1091, df = 37, ρ < 0.001) with a small to moderate degree of association (Cramer’s V = 0.269, ρ < 0.001, Eta = 0.269) and Day to Day Pain (χ^2^ = 59.835, *N* = 1091, df = 37, ρ = 0.010) with a small to moderate degree of association (Cramer’s V = 0.234, ρ = 0.010, Eta = 0.309). There was no significant difference for Favourite Colour. Figure 8 shows the percentage of colour selection for each question by Kinesiophobia classification. Comparing the permutations of Positive, Negative and Neutral Colours as outlined by Carruthers et al. [2,3] for sensitivity to Kinesiophobia classification showed the permutations had different sensitivities to Pain Catastrophising classification. Favourite Colour showed no sensitivity to Kinesiophobia classification. “Drawn to”, “Day-to-Day Mood” and “Day-to-day Pain” across permutations were sensitive to Kinesiophobia classification.

Augmenting with ML models showed that an NBC classified 73.1% of cases during training on the basis of Drawn To Colour. On testing, the best model was a CRT (10/2) model that had 25 terminal nodes and a tree depth of 5, and it used Day to Day Mood Permutations 0–7 and 8; Day to Day Mood; Day to Day Pain Permutations 0–7 and 8; Drawn To Colour; Drawn To Colour Permutations; and Favourite Colour Permutations 0–7 and 8.

### 3.3. Predictive Validity of the MCW

For a detailed description of the features from the recovery models, please see the Supplementary Materials “S3.3.1 Recovery Models with Features”.

#### 3.3.1. Recovery Models

ML models for predicting recovery status as USC/NR, PR and FR using all psychometric, demographic and MCW variables were calculated and compared for classification accuracy on “hold out” 80% training data and 20% testing data. The best model in training was an NBC model using automatic Gaussian binning of two continuous variables: Interval from Injury to Onboarding and VAS, which predicted outcomes with 71.5% accuracy (Macro AUC = Incalculable. Sn/Sp: FR= 66.3%/94.7%, PR = 62.2%/72.7%, USC/NR = 100%/72.3%). The best model in testing was a QUEST (5/2) model with four terminal nodes and a tree depth of 3, with 53.8% overall accuracy (Macro AUC = 0.616, Sn/Sp: FR= 70.9%/48.7%, PR = 0%/100%, USC/NR = 55.8%/69.9%).

ML models using psychometric test scores, age, optimism about recovery and Injury to Onboard Interval were calculated to predict recovery status. The best ML model in training was an Ex-CHAID (5/2) model with 28 terminal nodes and tree depth of 3, with 67.8% accuracy (Macro AUC = 0.828 Sn/Sp: FR= 81.5%/63.3%, PR = 42.3%/94.09%, USC/NR = 55.4%/84.6%).). The best ML Model in testing was a QUEST (10/2) model with six terminal nodes and a tree depth of 4, which predicted recovery with 56.8% accuracy (Macro AUC = 0.668, Sn/Sp: FR= 91.1%/26.6%, PR = 0%/100%, USC/NR = 29.5%/86.9%).

#### 3.3.2. Whiplash Associated Disorder Recovery Prediction Models

ML Models using all variables were calculated to predict recovery status from WAD. The best model in training was a CRT (10/2) model with 17 terminal nodes and a tree depth of 5, with 80.7% accuracy (Macro AUC= 0.809, Sn/Sp: FR= 70.9%/48.7%, PR = 0%/100%, USC/NR = 55.8%/69.9%). In testing, a QUEST (10/2) model was most successful, with two terminal nodes and a tree depth of 1, with 64.4% accuracy (Macro AUC = 0.570, Sn/Sp: FR= 93.9%/34.6%, PR = 0%/100%, USC/NR = 38.9%/90.2%).

#### 3.3.3. Back Injury Recovery Prediction Models

ML models using all variables were calculated to predict recovery status from BI. The best model in training was a CRT (10/2) model with 16 terminal nodes and a tree depth of 5, with 78% accuracy (Macro AUC = 0.811, Sn/Sp: FR= 86.3%/80.9%, PR = 54.7%/54.7%, USC/NR = 81.9%/84.7%). The best model on testing was a CHAID (10/2) model with five terminal nodes and a tree depth of 3, with 61.3% accuracy (Macro AUC = 0.661, Sn/Sp: FR= 91.6%/79.3%, PR = 0%/100%, USC/NR = 80.9%/75.9%).

#### 3.3.4. Shoulder Injury Recovery Prediction Models

ML models using all variables were calculated to predict recovery status in SI. The best model in training was a CRT (10/2) model with 18 terminal nodes and a tree depth of 5, with 81.7% accuracy (Macro AUC = 0.80, Sn/Sp: FR= 91.6%/ 79.3%, PR = 35.9%/97.3%, USC/NR =84.2%/90.7%). The best model on testing was a CHAID (5/2) model with 11 terminal nodes and a tree depth of 3, with 62.2% accuracy (Macro AUC = 0.63, Sn/Sp: FR= 86.0%/48.7%, PR = 20%/93.1%, USC/NR = 41.1%/88.7%).

#### 3.3.5. Neck Injury Recovery Prediction Models

ML Models using all variables were calculated to predict recovery status in NI. Given the small number of NI cases, no testing was conducted to retain power in training. The best model was an NBC that used the variables Interval from Date of Injury to Onboard, Kinesiophobia rating, VAS, Day to Day Mood and Favourite Colour, with 96.4% accuracy (Macro AUC = 0.971, Sn/Sp: FR= 96.6%/100.0%, PR = 91.7%/97.7%, USC/NR = 100%/97.3%).

#### 3.3.6. Comparison of Features in Recovery Prediction Models

General models across injury types emphasised pain and the Injury to Onboard interval as primary features for predicting recovery. However, WAD models relied on psychological variables first, while BI, SI and NI models relied on pain measurements first, emphasising the different dynamics at play in WAD.

## 4. Discussion

This study provides a comprehensive evaluation of the Manchester Colour Wheel (MCW) as a clinical tool for assessing psychological states of individuals undergoing rehabilitation from insured injuries. The findings underscore the MCW’s potential as a non-verbal, intuitive method for gauging emotional well-being, particularly in settings where traditional questionnaire-based assessments may fall short due to language barriers. The MCW demonstrated significant sensitivity to various psychological constructs, such as Anxiety, Depression and Pain Catastrophising. This aligns with earlier studies by Carruthers et al. (2010) [2], which indicated that individuals with mood disorders exhibit distinct colour preferences. In the present study, specific colours and their permutations were consistently associated with emotional states, with darker shades often linked to negative emotions such as Anxiety and Depression. The study leveraged Machine Learning (ML) algorithms to enhance the interpretation of the MCW. NBC and decision trees (e.g., CRT, QUEST) were employed to classify psychological states based on colour choices. The NBC models achieved a classification accuracy of up to 78% for Anxiety and Depression, suggesting that the integration of ML can significantly enhance the diagnostic precision of the MCW. This approach resonates with the work of Gozzi et al. (2024) [37], highlighting the integration of psychological and other data to tailor interventions more effectively. The study highlights the intricate relationship between psychological states and pain perception. It confirms that emotional distress can exacerbate pain experiences, a phenomenon well-documented by Puntillo et al. (2021) [24]. The MCW’s ability to capture these nuances through colour preferences offers a novel perspective in understanding how patients relate to their pain, thus providing a more holistic view of their recovery process. The MCW, when combined with ML, showed promise in predicting recovery outcomes. The study found that colour selections could be used alongside traditional psychometric measures to forecast recovery trajectories. For example, permutations of colour responses to specific questions (e.g., “Day-to-Day Mood”) were predictive of recovery status, indicating that the MCW can serve as a valuable prognostic tool in clinical settings.

Other features of recovery were identified through comparison within the research cohort. At onboarding, Surgery Withdrawal occurred more often in older participants than in those who made a full recovery or did not complete the program. People with SI were older than BI and WAD, perhaps reflecting the different dynamics of these injuries, with BI and WAD being injuries from incidents, while SI may be due to overuse and metabolic or age-related structural changes. Injuries that required surgery, or were deemed unsuitable for clinic, or did not commence the program when offered, and did not complete the treatment program, were significantly more painful injuries as measured by the VAS. These clients were significantly more stressed, hopeless, and had lower levels of interest than those who recovered, lending credence to the aphorism that “pain and stress are opposite sides of the same coin” [23]. This impression is furthered in that the best ML models using only the MCW relied on Day to Day Pain and Day to Day Mood ratings to predict recovery. It also emphasises the role of pre-morbid psychological factors in response to treatment, echoing the findings of other researchers [40,42,43], as no significant difference was found in the measurements of mood before and after treatment.

ML models based on the MCW and Vaegater et al.’s [46] questions had roughly equal accuracy for predicting outcomes of treatment. Different injuries had different recovery dynamics, and MCW and the psychometric measurements appear to have some unique construct value, as evidenced by the improvement in predictive accuracy for ML models using both sets of variables. Close examination of the variables in the ML models for each injury showed that the models for each injury used both the MCW and Vaegater et al.’s questions, and that these two methods of measuring psychological distress and pain have some overlap. There appears to be some unique contribution from Favorite Colour and Drawn To Colour, as these appear in models as often as other variables. For example, the NBC for Jensen Pain Classification relied on Drawn To Colour with some sensitivity to classifying 58.3% with mild pain, 75.8% of those with moderate pain and 12.9% with severe pain. This perhaps suggests that there is some common process or parallel process affecting the perception of pain and visual attraction. The MCW Permutations, clustering colours differentially as “positive”, “neutral” and “negative”, added to the accuracy of ML models. Similarly, different ML models relied on cutoffs on psychometric ratings, as well as treating these measurements as continuous variables. This suggests that these classifying cutoffs are clinically meaningful and contribute to the construct validity of these measurements.

The performance of ML models in training and testing for this kind of health data appears to reflect greater training accuracy in models that are more complex, with simpler models like QUEST better maintaining accuracy in testing of prediction. This may reflect that the testing case series length was not big enough for more complex models to reach their baseline efficacy or that more complex DT models were overfitting. ML models based on the rating of the four questions on the MCW had weak capacity to classify pain (49.2%) but had good classification of Anxiety (75.6%) and Depression (70.3%), and moderate classification for Pain Catastrophisation (69.4%), Stress (68.5%), and Kinesiophobia (62.7%) on testing. The most successful models on testing were CRT models, suggesting that these were not overfitting.

Limitations to the study include that the intervention period was relatively brief, a maximum of 12 weeks, and long-term outcomes cannot be inferred from the data. There were also overlaps in colour selections in response to questions on the MCW by people classified with different types of psychological distress. It is possible that this reflects non-verbal links between emotions, a general distress, and there is literature that shows overlap between somatic, depressed and anxious distress, with greater overlap in non-clinical populations than in primary care [62]. There is greater overlap of anxiety and depression in the acute phase, with greater differentiation of symptoms later in the course of symptom expression [63]. Future research is required to distinguish between colour selection and types of distress, especially as conditions evolve over time. Another limitation was that only four questions were used to elicit ratings on the MCW, and all these questions had some discriminant value in classification and prediction problems; however, systematic testing of other prompting questions using the current research methodology may improve accuracy. Further research is required to see what other questions and suggestions might improve the discriminant value of the MCW. While a conventional method in testing ML models, another limitation was the 80% training and 20% testing protocol, and further cross-validation with a sufficiently large and unique data set is required. Another limitation was that this research relied on a natural clinical sample, and there were markedly different numbers of people in the different injury groups and recovery status of these groups. In particular, there were very few people in the NI group. Greater equivalence between comparison groups would improve the quantification of the contribution of the test constructs to the classification and prediction problems they were applied to. Another limitation was that there was no information relating to the cultural backgrounds of clients, and future research could compare possible cultural contributions to colour preference and the expression of pain [64]. The precise ethnic and cultural constitution of the sample could not be estimated. The MCW’s sensitivity to the different test factors measured psychometrically supports its potential utility as a non-verbal assessment method. Further research is required using valid translations of questionnaires to assist in clarifying the construct validity of the MCW. The MCW may access right-hemisphere-mediated aspects of the injury experience that are less accessible through traditional verbal measures; however, only some inferences can be made from apparent correlation on the basis of the current research. Further research is required to clarify the process of making a rating on the MCW, and possible avenues of inquiry could be FMRI scanning during rating, TMS interference of left hemisphere processing during rating or split-brain study type protocols to see if there is some unique process for MCW type ratings versus questionnaire type ratings. Other future research would be to apply more sophisticated Machine Learning methods, such as ensemble models like extreme gradient boosting regression and random forest, to larger data sets to see what other classifications and predictions can be made with MCW and psychometric data.

Other future research could use the MCW with clients undergoing EMDR to rate distress and body sensations with associated colours during the processing phase of therapy to see if there are constant relationships between colours and emotional processing. This could also show how psychological complexes and emotions can be changed with appropriate therapy. An important finding is that psychological factors appear to remain relatively unchanged despite treatment in an interdisciplinary clinic. It may be that not enough treatment was provided during the treatment period to address the psychological factors, or that the treatment was not as efficacious as expected. Future research could address improving psychological treatment protocols or making the treatment of psychological factors primary to the treatment of physical factors, which may improve recovery rates. This paper represents a first step towards validating the MCW as an alternative or adjunct to questionnaires, especially when augmented with ML.

## 5. Conclusions

In conclusion, this study provides evidence for the clinical utility of the MCW in assessing psychological adjustment to injury and predicting recovery outcomes. The tool’s sensitivity to various psychological states and its contribution to improved predictive modelling suggest it may be a valuable addition to existing clinical assessment protocols. Future research should focus on validating these findings in diverse populations and further exploring the practical implementation of the MCW and ML in clinical settings.

## Figures and Tables

**Table 1:** Outcomes, means and standard deviations at onboarding and discharge.

Status	*N* (%)	1 ^a^	2	3	4	5	6	7	8	9	10
USC	39 (3.37)	47.43 (12.73)	33.79 (45.59)	57.7 (25.4)	6.28 (2.63)	7.13 (2.70)	6.08 (3.36)	5.87 (3.07)	5.85 (3.44)	5.51 (3.54)	41.0/41.0/18.0
SW	38 (3.7)	49.57 (13.00)	14.29 (19.64)	62.4 (19.9)	5.55 (3.49)	6.11 (3.29)	5.55 (3.19)	5.29 (3.12)	5.58 (2.83)	6.24 (3.07)	7.9/71.1/21.0
DNC	153 (13.9)	40.97 (13.33)	13.27 (18.74)	59.2 (22.8)	6.21 (2.93)	6.68 (2.63)	6.24 (2.96)	5.84 (2.73)	5.72 (3.05)	6.04 (3.09)	30.1/66.0/3.9
DNCP	97 (8.9)	40.92 (12.52)	18.33 (28.61)	65.2 (19.3)	6.54 (3.01)	7.46 (2.38)	6.79 (2.97)	6.51 (2.88)	6.98 (2.81)	6.80 (2.75)	23.7/70.1/6.2
AW	5 (0.5)	55.40 (7.37)	7.40 (3.78)	44.0 (20.7)	4.20 (4.03)	5.20 (3.11)	4.40 (4.04)	5.20 (4.09)	3.60 (2.41)	4.40 (2.41)	40.0/60.0/0.0
NR	17 (1.6)	43.53 (13.16)	33.79 (45.99)	61.8 (1.78)	6.06 (2.70)	6.94 (3.01)	7.06 (2.82)	6.18 (2.86)	7.06 (2.25)	7.94 (2.28)	29.7/58.5/11.8
NR Disc.				62.5 (18.5)	5.58 (3.73)	6.33 (2.81)	6.17 (3.09)	5.17 (2.83)	6.00 (2.29)	6.00 (3.41)	
PR	192 (17.5)	44.55 (12.86)	18.71 (25.00)	58.9 (18.6)	5.82 (2.96)	6.51 (2.56)	5.90 (2.97)	5.45 (2.68)	5.88 (2.72)	5.49 (3.07)	28.1/66.1/5.8
PR Disc.				51.9 (18.2)	5.32 (2.89)	5.76 (2.41)	5.38 (2.78)	4.99 (2.73)	5.41 (2.49)	5.29 (2.94)	
FR	555 (50.5)	41.34 (13.27)	16.76 (35.00)	42.9 (23.0)	4.78 (3.11)	5.38 (2.91)	4.59 (3.19)	4.36 (3.00)	4.27 (3.10)	4.36 (3.13)	53.7/42.5/3.8
FR Disc.				41.3 (23.5)	4.21 (3.09)	4.73 (2.91)	4.18 (3.05)	4.10 (2.99)	4.05 (3.03)	4.32 (3.13)	
Cum.	1096 (100)	42.36 (13.28)	17.51 (31.34)	51.5 (23.6)	5.42 (3.12)	6.06 (2.88)	5.37 (3.22)	5.07 (3.00)	5.13 (3.13)	5.17 (3.21)	100/100/100

1 = Age, 2 = Weeks from Injury to Onboard Interval, 3 = Visual Analogue Pain Scale (VAS, mm), 4 = Anxiety rating, 5 = Stress rating, 6 = Hopelessness rating (Depression Item 1), 7 = Low Interest rating (Depression Item 2), 8 = Pain Catastrophising rating, 9 = Kinesiophobia rating, 10 = optimism about recovery (Yes%/Unsure%/No%). “Disc.” = score at discharge, USC = Unsuitable for Clinic, SW = Surgical Withdrawals, DNC = Did Not Commence, DNCP = Did Not Complete, AW = Administrative Withdrawal, NR = No Recovery, PR = Partial Recovery, FR = Full Recovery. ^a^ Date of birth was only available for 1015 participants.

**Table 2:** Means and standard deviations (SDs) of Whiplash Associated Disorder (WAD), Neck Injury (NI), Back Injury (BI), Shoulder Injury (SI) and Cumulatively (Cum.).

Injury	*N*	%	1 ^a^	2	3	4	5	6	7	8	9	10
WAD	260	23.72%	40.06 (13.51)	7.05 (4.38)	53.8 (24.1)	6.53 (2.66)	5.99 (3.08)	5.83 (3.15)	5.17 (2.95)	5.18 (3.29)	5.15 (3.33)	164.78 (16.62)
NI	55	5.02%	25.36 (11.40)	25.36 (59.03)	53.5 (21.4)	6.85 (2.60)	6.85 (2.60)	5.91 (3.11)	5.71 (3.13)	6.11 (3.22)	5.69 (3.21)	242.20 (38.80)
BI	364	33.21%	41.30 (12.67)	21.20 (36.67)	51.9 (23.1)	6.18 (2.82)	5.47 (3.07)	5.56 (3.20)	5.14 (3.00)	5.22 (3.09)	5.21 (3.15)	166.33 (18.82)
SI	391	35.75%	45.01 (13.49)	20.69 (27.61)	49.0 (23.7)	5.47 (3.03)	6.53 (2.66)	4.78 (3.19)	4.82 (3.00)	4.88 (3.02)	5.08 (3.17)	218.54 (16.11)
Cum.	1096	100	42.41 (13.26)	17.53 (31.37)	51.4 (23.6)	6.05 (2.88)	6.05 (2.88)	5.37 (3.22)	5.06 (3.00)	5.13 (3.13)	5.17 (3.21)	188.77 (18.57)

1 = Age, 2 = Weeks from Injury to Onboard Interval, 3 = Visual Analogue Pain Scale (VAS, mm), 4 = Anxiety rating, 5 = Stress rating, 6 = Hopelessness rating (Depression Item 1), 7 = Low Interest rating (Depression Item 2), 8 = Pain Catastrophising rating, 9 = Kinesiophobia rating, 10 = optimism about recovery (Yes%/Unsure%/No%). “Disc.” = score at discharge, USC = Unsuitable for Clinic, SW = Surgical Withdrawals, DNC = Did Not Commence, DNCP = Did Not Complete, AW = Administrative Withdrawal, NR = No Recovery, PR = Partial Recovery, FR = Full Recovery. ^a^ Date of birth was only available for 1015 participants.

**Table 3:** Outcomes by injury type.

Status	USC (%)	DNC (%)	DNCP (%)	NR (%)	PR (%)	FR (%)
WAD	10 (2.75)	52 (14.28)	21 (5.76)	5 (1.38)	46 (12.67)	150 (41.21)
NI	5 (18.18)	4 (7.27)	6 (10.90)	3 (5.46)	12 (21.81)	25 (45.45)
BI	18 (4.97)	51 (14.01)	36 (9.90)	5 (1.37)	81 (22.25)	171 (46.98)
SI	44 (11.28)	46 (11.76)	34 (8.70)	4 (1.02)	53 (13.55)	209 (53.45)
Cum.	77	153	97	17	192	555

WAD = Whiplash Associated Disorder, NI = Neck Injury, BI = Back Injury, SI = Shoulder Injury, “Cum.” = Cumulatively, USC = Unsuitable for Clinic, DNC = Did Not Commence, DNCP = Did Not Complete Treatment, NR = No Recovery, PR = Partial Recovery, FR = Full Recovery.

**Table 4:** Percentage without/percentage with/overall percentage classified by Machine Learning models built on MCW in response to 4-question ratings on 80% training and 20% “test” data for Anxiety, Stress, Depression, Pain Catastrophisation (Pain C.) and Kinesiophobia classification (Kines C.).

Classifier (Min. Parent/Child) and Test Trial	Anxiety	Stress	Depress.	Pain C.	Kines C.
CHAID (10/2)	38.6/85.3/69.4	69.3/61.2/65.2	79.4/46.7/66.3	94.9/18.5/65.7	6.4/96.2/62.8
Test	28.6/80.0/60.4	60.2/58.4/59.3	77.1/32.1/61.9	95.5/17.2/63.4	7.5/96.9/62.7
CHAID (5/2)	50.6/79.7/69.6	72.4/53.9/62.2	88.1/32.8/66.6	85.5/39.8/67.7	45.3/76.1/64.4
Test	38.9/70.9/59.7	74.8/53.3/65.2	86.3/26.8/63.4	83.8/32.1/64.1	37.0/70.3/58.5
Ex-CHAID (10/2)	0.0/100/64.8	55.5/71.9/63.7	79.8/43.1/65.1	87.9/37.3/69.1	0.9/99.8/63.3
Test	0.0/100/65.5	51.0/61.9/56.9	85.2/38.6/69.8	84.2/30.2/59.5	0.0/98.5/60.0
Ex-CHAID (5/2)	26.8/91.7/68.3	72.9/50.8/61.7	94.3/21.1/66.1	87.3/32.6/66.4	37.3/80.4/64.4
Test	23.0/88.8/68.7	73.1/61.8/67.3	88.5/25.3/63.5	89.6/28.3/64.6	27.9/77.9/56.5
CRT (10/2)	61.4/77.8/71.9	67.7/63.7/70.0	87.3/54.3/74.8	84.1/59.9/74.7	50.6/79.5/68.5
Test	66.7/79.5/75.6	77.5/59.2/68.5	77.4/45.3/63.7	75.4/41.9/62.3	37.2/67.3/57.0
CRT (5/2)	50.2/85.9/74.0	79.3/63.7/71.4	85.1/55.1/73.3	83.4/57.8/73.6	42.4/82.6/67.2
Test	33.7/84.7/63.4	64.5/61/4/62.9	83.5/73.3/70.3	78.1/41.5/63.2	19.7/79.5/59.6
QUEST (10/2)	11.6/97.2/68.0	64.2/67.1/65.8	87.6/26.7/64.0	89.5/23.6/63.6	7.4/97.1/64.1
Test	9.0/97.7/64.4	59.7/60.2/59.9	86.8/14.0/58.6	92.8/28.2/69.4	6.0/98.4/61.4
QUEST (5/2)	19.9/93.5/69.1	65.2/64.6/64.9	86.4/39.9/68.1	89.3/22.6/63.4	0.0/100/62.6
Test	14.6/87.1/56.8	62.8/64.1/63.5	82.7/37.5/66.2	89.7/16.7/61.8	0/100/62.7
NBC	45.9/94.0/78.0	73.3/82.1/77.7	75.6/60.1/73.4	87.2/46.7/72.0	46.2/86.0/69.4
Test	33.7/85.8/67.4	65.8/63.6/60.3	68.1/60.4/65.1	72.8/33.9/58.2	27.3/77.8/59.5

## Data Availability

The data presented in this study are available on request from the corresponding author. As the data were collected by Navigator Group Pty Ltd. as part of its business, it reserves the right to determine access to the data and the intent of the use of the data.

## Supplementary Materials

The following supporting information can be downloaded at: https://www.mdpi.com/article/10.3390/jcm15010075/s1, Paragraphs S3.3.1–S3.3.5 listing all the variables utilised by the ML models.

## Abbreviations

The following abbreviations are used in this manuscript:

AI	Artificial Intelligence
ARC	Active Recovery Clinics
AW	Administrative Withdrawal
BI	Back Injury
CHAID	Chi-Square Automatic Interaction Detector
CRT	Classification and Regression Tree Model
DI	Digital Intervention
DNC	Did Not Commence
DNCP	Did Not Complete
EMDR	Eye Movement Desensitisation and Reprocessing Therapy
fMRI	Functional Magnetic Resonance Imaging
FR	Full Recovery
MCW	Manchester Colour Wheel
ML	Machine Learning
MVC	Motor Vehicle Crash
NBC	Naïve Bayesian Classifier
NI	Neck Injury
NR	No Recovery
PR	Partial Recovery
PTSD	Posttraumatic Stress Disorder
QUEST	Quick Unbiased Efficient Statistical Tree
SD	Standard Deviation
SI	Shoulder Injury
SW	Surgical Withdrawals
USC	Unsuitable for Clinic
VAS	Visual Analogue Pain Scale
WAD	Whiplash Associated Disorder
