# Supplementary material for: The Clinical Significance of the Manchester Colour Wheel in a Sample of People Treated for Insured Injuries
Source: J Clin Med. 2025 Dec 22;15(1):75. doi: 10.3390/jcm15010075 (PMC12786711; doi:10.3390/jcm15010075)
Supplement: Supplementary file 1 [file jcm-15-00075-s001.zip › jcm-3999617-supplementary.pdf]

S3.3.1 Recovery Models with Features

ML models for predicting recovery status as USC/NR, PR, and FR using all psychometric, demographic and MCW variables were calculated and compared for classification accuracy on 80% training data and 20% testing data. The best model in training was an NBC model using automatic Gaussian binning of two continuous variables Interval from Injury to Onboarding and VAS which predicted outcomes with 71.5% accuracy. The best model in testing was a QUEST (5/2) model with 4 terminal nodes, a tree depth of 3, and used in order Jensen Pain Classification, VAS, pain catastrophisation question, optimism about recovery, Vaegter et al's anxiety question, day to day pain on the MCW, hopelessness question, and Day to Day Pain Permutations 0, 1, 4, 2, and 3 as variables and predicting outcomes with 53.8% overall accuracy.

ML models using psychometric test scores, age, optimism about recovery and Injury to Onboard Interval were calculated to predict recovery status. The best ML model in training was an Ex-CHAID (5/2) model with 28 terminal nodes and tree depth of 3, which used Interval from Injury to Onboard, Kinesiophobia classification, Kinesiophobia rating, optimism about recovery, pain catastrophisation rating, depressed classification, VAS, and hopelessness classification, which predicted recovery with 67.8% accuracy. The best ML Model in testing was a QUEST (10/2) model with 6 terminal nodes and tree depth of 4, which relied on Jensen Pain Classification, VAS, pain catastrophisation rating, stress rating, optimism about recovery, sense of hopelessness rating, stressed classification, depression classification, anxiety classification, kinesophobia rating, and low interest rating which predicted recovery with 56.8% accuracy.

S3.3.2 Whiplash Associated Disorder Recovery Prediction Models

ML Models using all variables were calculated to predict recovery status from WAD. The best model in training was a CRT (10/2) model with 17 terminal nodes and a tree depth of 5, and used the variables in order: hopelessness rating, little interest rating, feeling anxious rating, anxiety classification, pain catastrophisation rating, stress rating, VAS, Boonstra Pain Function Classification, Boonstra Pain Cluster Classification, Jensen Pain Classification, Day to Day Pain Permutation 7 & 8, Day to Day Mood Permutation 0, Day to Day Mood Permutation 4, Day to Day Pain Permutation 2, Day to Day Pain Permutation 5 & 6, Kinesiophobia Classification, Day to Day Pain Permutation 3, Day to Day Pain Permutation 1, Day to Day Pain Permutation 4, Day to Day Mood Permutation 3, Day to Day Pain Permutation 1, Interval From Injury to Onboard, Depressed Classification, Day to Day Mood Permutation 5 &

6, Sum of Colour Wheel Ratings, Favourite Colour, Drawn to Colour, Optimism About Recovery, Day to Day Pain, Favourite Colour Permutation 5& 6 and 7 & 8, Favourite Colour Permutation 1, Day to Day Mood, Favourite Colour Permutation 0, Favourite Colour Permutation 4, Drawn To Permutation 2, Drawn To Permutation 3, Favourite Colour Permutation 2, Favourite Colour Permutation 4, Drawn to Permutation 2, Drawn to Permutation 3, Favourite Colour Permutation 2, Drawn to Permutation 0, Drawn to Permutation 4, Drawn to Permutation 5 & 6, Drawn to Permutation 7 & 8, and Pain Catastophization Classification with 80.7% accuracy. In testing a QUEST (10/2) model was most successful testing with a 2 terminal nodes and a tree depth of 1, using variables of Jensen Pain Classification, VAS, Boonstra Pain Classification Function, Interval From Date of Injury to Onboard, and Drawn to Colour with 64.4% accuracy.

S3.3.3 Back Injury Recovery Prediction Models

ML models using all variables were calculated to predict recovery status from BI. The best model in training was a CRT (10/2) model with 16 terminal nodes and a tree depth of 5 using variables in order of VAS, Jensen Pain Classification, pain catastrophization rating, little interest rating, Boonstra Pain Classification Function, Boonstra Pain Classification Cluster, hopelessness rating, stress rating, kinesiophobia rating, Interval From Date of Injury to Onboard, anxious rating, depressed classification, optimism about recovery, Favourite Colour, kinesiophobia classification, Drawn To Permutation 2, Day to Day Mood, Drawn To Permutation 1, Drawn to Permutation 0, Drawn to Permutation 3, Drawn to Premutation 4, stressed classification, Drawn to Colour, Favourite Colour Permutation 2, Drawn to Colour Permutation 5 & 6, Drawn to Colour Permutation 7 & 8, anxiety classification, Drawn to Colour Permutation 2, Drawn to Colour Permutation 0. Drawn to Colour Permutation 3, Drawn to Colour Permutation 4, Drawn to Colour Permutation 1, Pain Catastrophization Classification, Favourite Colour Permutation 0, Favourite Colour Permutation 4, Day to Day Pain Permutation 1, Day to Day Pain, Day to Day Pain Permutation 2, Favourite Colour Permutation 1, Favourite Colour Permutation 5 & 6, Day to Day Pain Permutation 5 & 6, Day to Day Pain Permutation 0, Day to Day Pain Permutation 4, Day to Day Pain Permutation 3, Day to Day Pain Permutation 7 & 8, Favourite Colour Permutation 3 with 78% accuracy. The best model on testing was a QUEST (10/2) model with 5 terminal nodes and a tree depth of 3, using variables VAS, Jensen Pain Classification, Boonstra Pain Classification Function, stress rating, pain catastrophizing rating, Day to Day Pain, Day to Day Pain Permutation 2, Day to Day Pain Permutation 1, Day to Day Pain Permutation 0, Day to Day Pain Permutation 4, Favourite Colour, Interval from Date of Injury to Onboard, Drawn To Colour, Day

to Day Mood, pain catastrophizing classification, kinesiophobia rating, hopelessness rating, little interest rating with 59.7% accuracy.

S3.3.4 Shoulder Injury Recovery Prediction Models

ML models using all variables were calculated to predict recovery stutus in SI. The best model in training was a CRT (10/2) model with 18 terminal nodes and a tree depth of 5 and used the variables in order of VAS Total , Boonsta Pain Classification Function, Boonstra Pain Classification Cluster, Jensen Pain Classification, pain catastrophization rating, stress rating, kinesiophobia rating, little interest rating, anxiety rating, Day to Day Pain Permutation 5 & 6, kinesiophobia classification, hopelessness rating, anxiety rating, Day to Day Pain, Favourite Colour, Day to Day Mood, Drawn to Colour Permutation 7 & 8, Favourite Colour Permutation 5 & 6, Drawn to Colour, Optimism, Drawn To Permutation 2, Interval from Date of Injury to Onboard, Drawn to Permutation 1, Drawn to Permutation 3, Drawn to Permutation 5 & 6, Drawn To Permutation 0, Drawn to Permutation 4, depressed classification, Day to Day Pain Permutation 1, Day to Day Pain Permutation 2, stressed classification, Day to Day Mood Permutation 1, Day to Day Pain Permutation 3, Day to Day Mood Permutation 0, Day to Day Pain Permutation 4, Day to Day Mood Permutation 5 & 6, Day to Day Mood Permutation 3, Favourite Colour Permutation 2, pain catastrophizing classification, Day to Day Mood Permutation 7 & 8, Favourite Colour Permutation 7 & 8, Favourite Colour Permutation 0, and Favourite Colour Permutation 4 with 81.7% accuracy. The best model in testing a CHAID (5/2) model with 11 terminal nodes and a tree depth of 3, using variables in order of Boonstra Pain Classification Function, pain catastrophizing rating, Day to Day Pain Permutation 7 & 8, Drawn To Permutation 7 & 8, kinesiophobia classification, Day to Day Mood Permutation 5 & 6, and Favourite Colour Permutation 3 with 62.2% accuracy.

S3.3.5 Neck Injury Recovery Prediction Models

ML Models using all variables were calculated to predict recovery status in NI. Given the small number of NI cases, no testing was conducted to retain power in training. The best model was a NBC which used variables Interval from Date of Injury to Onboard, kinesiophobia rating, VAS, Day to Day Mood and Favourite Colour with 96.4% accuracy.
